# Preterm birth update in Australasia: A report of the international symposium of Preterm Birth International Collaborative-Australasia branch

**DOI:** 10.3389/fped.2022.903546

**Published:** 2022-07-25

**Authors:** Chong Qiao, Ramkumar Menon, Ki Hoon Ahn, Shunji Suzuki, Pallavi Kshetrapal, Harry Michael Georgiou, Sam Mesiano, Nanbert Zhong

**Affiliations:** ^1^Department of Obstetrics and Gynecology, Shengjing Hospital of China Medical University, Shenyang, China; ^2^Division of Basic Science and Translational Research, Department of Obstetrics and Gynecology/Cell Biology, The University of Texas Medical Branch at Galveston, Galveston, TX, United States; ^3^Preterm Birth International Collaborative, South Burlington, VT, United States; ^4^Department of Obstetrics and Gynecology, Korea University College of Medicine, Korea University Anam Hospital, Seoul, South Korea; ^5^Department of Obstetrics and Gynecology, Nippon Medical School, Tokyo, Japan; ^6^Translational Health Science and Technology Institute, National Capital Region Biotech Science Cluster, Faridabad, India; ^7^Department of Obstetrics and Gynecology, University of Melbourne, Parkville, VIC, Australia; ^8^Department of Reproductive Biology, Case Western Reserve University, Cleveland, OH, United States; ^9^Department of Obstetrics and Gynecology, University Hospitals of Cleveland, Cleveland, OH, United States; ^10^New York State Institute for Basic Research in Developmental Disabilities, New York, NY, United States

**Keywords:** symposium report, preterm birth, PREBIC, Australia, maternal and children's health

## Abstract

Preterm birth (PTB) is one of the most important problems that pose dilemmas for both the obstetrician and neonatologist, placing a heavy burden psychologically and financially on the families involved, and triggering high socio-economic costs to the public healthcare. The rate of PTB in Asian countries has been ranked at top globally. To reduce the PTB rate, to promote the prevention and intervention for PTB, and to better understand the pathophysiology underlying PTB, the Preterm Birth International Collaborative Australia branch (PREBIC-AA) was launched in 2017. A series scientific activities including organizing annual research symposiums has been planned and organized among Australasian countries. Here we briefly updated the current progress in clinical management and translational research on PTB in Australasian countries that have been participated in PREBIC-AA.

## Introduction

### History of the Preterm Birth International Collaborative Australasia branch

The Preterm Birth International Collaborative (PREBIC) is composed of leading researchers, clinicians, and clinical academics from Europe, Asia, North America, South America, and Australia, and its mission is to improve the quality of life of preterm infants and reduce the birth defects of children. Facilitated by Dr. Nanbert Zhong, who was the president of PREBIC at the time, the PREBIC-Australasia (PREBIC-AA) branch was launched on October 27, 2017, at the First PREBIC-Australasia Symposium, held in Haikou, Hainan Province (1, 2). In the following years, the Annual Meetings and Symposiums of PREBIC-AA were held in different cities in Asia and Australia (Showed in Table 1). However, the COVID-19 pandemic disrupted the schedule, resulted in the suspension of the conference. For the continuity of the scientific cause that the nations and groups have come together and to keep the “flame” of PREBIC-AA activities, the council restarted the symposium on February 26, 2021 in a virtual webinar format (3).

**Table 1:** History of PREBIC-AA symposium.

**Date**	**City**	**Topic**	**Board members**
2017-10-27	Haikou, China	Improve the quality of the life of preterm infants and reduce the birth defects of children.	President: Nanbert Zhong, M.D., Ph.D., New York State Institute for Basic Research in Developmental Disabilities, USA
2018-08-30	Seoul, Korea	Mechanism, prediction, prevention and treatment of preterm birth, epidemiology and other aspects.	President: Young Ju Kim, M.D., Ewha Women's University, Korea
2019-06-19	Shenyang, China	Hot and difficult issues on the occurrence, development, molecular pathological mechanism, clinical intervention and international cooperation of preterm birth.	President: Caixia Liu, M.D., Shengjing Hospital of China Medical University,China
2021-02-26	Webinar symposium	The Coronavirus Disease 2019 (COVID-19) pandemic and preterm birth.	President: Jun Takeda, M.D., Ph.D., Juntendo University Faculty of Medicine, Japan
2021-12-18	Webinar symposium	Current situation and perspectives of preterm birth in Australasia.	President: Chong Qiao, M.D., Ph.D., Shengjing Hospital of China Medical University,China

### PREBIC-AA symposium 2021

While further COVID disruptions continued throughout 2021, the International Symposium of PREBIC-AA resumed as a virtual meeting on December 18th, 2021. More than 100 participants attended the meeting from China, Japan, Korea, Australia, India, and the United States. The symposium was a mix of clinical physicians, experts and enthusiasts who are working on preterm birth and related complications. Informative lectures toward understanding of the etiology and biological markers of preterm birth, respectively were invited. Topics also covered the latest research progress made toward improving our knowledge on the fundamental basic research of preterm birth, augmented by multi-disciplinary fields like obstetrics, neonatology, pediatrics, perinatology, genetics, neurology, immunology, imaging, radiology, pathology, laboratory medicine, cell biology, molecular biology, and bioinformatics, to help us move forward toward finding tangible solutions as translational outputs for this unmet problem.

### Burden of preterm birth in Australasia

#### Prevalence of PTB in Asian countries

Preterm birth, defined as delivery before 37 weeks' gestation, is the leading cause of death in children younger than 5 years worldwide (4). Despite the medical technology becoming more advanced in the last few decades, preterm birth rate had continued to increase from 9.8% in 2000 to 10.6% in 2014, which equates to an estimated 14.84 million live preterm births. More than half (52.9%) of these preterm births took place in Asia. The authoritative statistics of “Top 10 countries for the number of preterm births in 2014” showed that four of the top five countries are from Asia and they accounted for 6.6 million (44.6%) of preterm births globally. They were, respectively, India (23.4%), China (7.8%), Bangladesh (4.0%), Indonesia (3.5%) (5).

#### National reports on PTB prevalence

Besides the accurate reports on prevalence of preterm birth worldwide, an increasing number of countries have their own detailed domestic research on PTB.

##### China

Researchers from China showed an increase in the preterm rate by 1.1% per year from 1990 to 2016 *via* a meta-analysis (6). Another report estimated an increase of 1.3% per year from 2012 to 2018 (7). These data indicated that with the popularization of two-child policy, the preterm birth rate is accelerating.

##### Japan

Similarly, the PTB rate of Japan has been increasing steadily from 1980 to 2000 but has remained constant after that. Although Japan has failed to further reduce PTB rate, the latest confirmed vital statistics of the population are those of 2016, and according to that, there were 976,978 live births including 54,594 PTBs (5.6%) (8).

##### India

There have been isolated regional studies on PTB from India that aimed to assess the maternal risk factors but were conducted on a smaller sample size (9, 10). Garbh-Ini pregnancy cohort has reported a high rate of PTB births (14.9% among 1,662 live births) based on a interim analysis of the cohort (11). Interestingly, similar statistics on PTB rates (14.98 %) had been reported in a study from a total of 2,611 documented live deliveries from the West of India (12).

##### Korea

According to a hospital-based retrospective cohort study of 3,554 singleton pregnancies (13), the preterm rate for women with underweight, normal weight and overweight are 7.0, 8.7, 13.0%. The PTB rate has decreased for decades but remains at a high level, which in turn brings heavy burden to society. The Korean Neonatal Network (14) reported that the survival rate of very low birth weight infants increased from 83.0% in the 2000's to 85.7% in the 2010's. There was also a significant increase in the survival rate of extremely low birth weight infants from 66.1 to 70.7%.

##### Australia

There has been little change in the proportion of preterm birth over the past decade, ranging from 8.2% in 2009 to 8.6% (25,933) in 2019. Babies born with low birth weight ranged from 6.2% in 2009 to 6.6% (19,982) in 2019 (15, 16).

#### Psychological and financial burden of PTB

**Figure 1:**
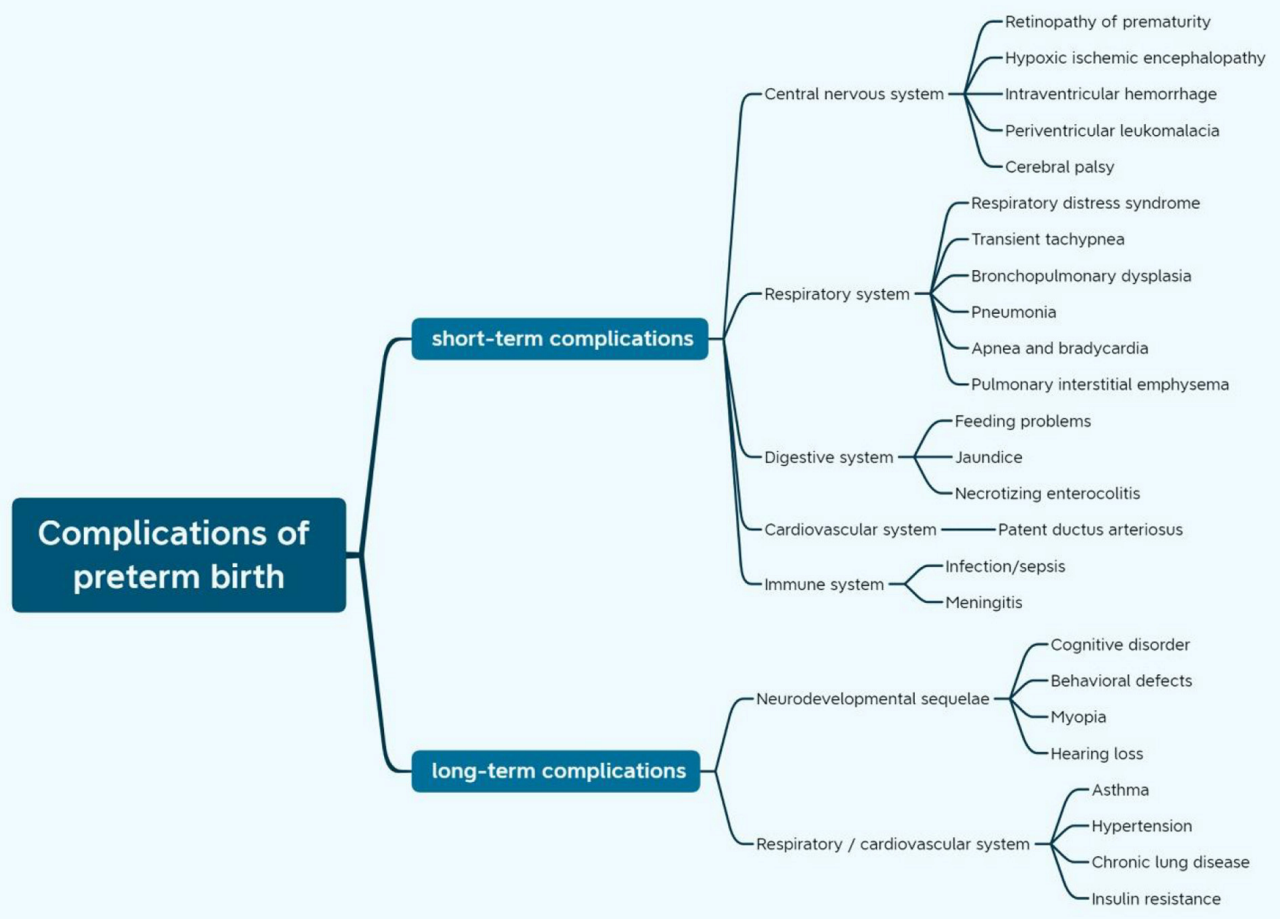
Complications of preterm birth.

Extremely preterm birth can lead to neonatal death, even if those who survive will be involved in the greater risk of a variety of potential consequences in all organ systems for the physical, neurodevelopmental, and behavioral development of these children (Figure 1) (17–22). All these complications can result in long-term effects, which in turn, place a heavy psychological and financial burden on the families involved and trigger high socio-economic costs to the health system (18, 23–26).

## Updated current progress: Lectures on special topics

### Placental omics and preterm birth

An omics-based “Two-Hit” hypothesis was introduced by Dr. Nanbert Zhong. The “1st-Hit” is genetic predisposition determined by the DNA sequence that can be studied with genomic approach. Gene-environmental interaction presents as the “2nd-Hit” that may result in epigenetic or epigenomic alterations. Metabolomic and microbiomic factors are the common environmental factors, which may impact gene transcription and result in transcriptomic and proteomic changes. To profile integrated “omics” may help investigate the pathogenic mechanism underlying spontaneous preterm birth. Proteomic study on preterm premature rupture of membrane (pPROM) demonstrated that extracellular matrix has been altered in preterm fetal membranes due to proteolysis. Metabolism was also altered in preterm fetal membranes. The molecular changes in the fetal membranes provided a significant molecular signature for pPROM in preterm syndrome (27), although there was no definitive gene/locus could be identified to associate with PTB (28).

### Preterm birth presents a complex phenotype

It has been difficult to fully understand preterm birth because of its heterogeneities in the definition, pathophysiologic heterogeneities, etiologic heterogeneities, genetic heterogeneities, and environmental heterogeneities. Professor Ramkumar Menon emphasized the needs for international collaborative efforts to reduce the incidence of PTB. A meta-analysis including 217 studies on maternal biomarkers of preterm birth from 1965 to 2010 shows that a total of 116 different biomarkers have been reported in spontaneous preterm (29). The huge disparity in many fundamental aspects of published reports creates heterogeneity making it difficult to combine these studies for a meta-analysis to assess their risk association. Similar data from systematic reviews of multiplex and proteomics-based biomarkers studies. No reliable biomarkers that will identify high-risk pregnancies for preterm labor were identified (30–33).

### The role of cervix in preterm labor

Although spontaneous preterm birth is a complex phenotype and multifactorial adverse pregnancy, cervical dysfunction plays an important role in the pathology. The cervix is generally composed of collagen-based extracellular matrix and some fibroblasts, muscle cells, glandular cells, vascular cells, and immune cells, but the proximal part has more muscle cells than the distal part. The cervical canal runs along its length and connects the cavity of the body of the uterus with the lumen of the vagina. The openings are known as the internal os and external os (or external orifice of the uterus), respectively. The internal os of the cervix is different from the nearby tissues in histological structure and imaging location. Some scholars predict that muscle cells near the internal os would function as sphincters and play a role in the funneling phenomenon seen in ultrasound (34). Numerous studies have shown that the composition and metabolism of collagen in the cervix are different from those of the normal group in the preterm labor group (35). This interaction with the extracellular matrix around the muscle cell affects the function of the muscle cell, and sex hormones seem to play an important role in the structure and function of the muscle cell and the extracellular matrix.

### Preterm birth and placental immunity

Preterm birth is clinically divided into spontaneous preterm delivery and iatrogenic preterm delivery. Infection and infection-derived activation of the inflammatory response are thought to be the leading risk factor of spontaneous preterm birth. The molecular triggers and mechanisms underlying the activation of immune pathways associated with the induction of preterm birth remain poorly understood. Innate immune cells [neutrophils, macrophage, mast, dendritic cells (DCs)] and adaptive immune cells (placental T cell, NKT cells, γδ T cells, B cells) are jointly involved in preterm birth. Similar to term birth, idiopathic preterm birth is preceded by selective accumulation of decidual macrophages, the depletion of which protects pregnant mice from LPS-induced preterm birth (36). Mast cells are also important innate immune effector cells during late gestation and labor due to their secretion of long-term modulator cytokines, mediators, and surface molecules. However, mast cells maybe not be the sole leukocyte recruiters, and the pro-inflammatory cascade can be upregulated by other subpopulations even in the absence of mast cells (37).

## Updated current progress: Regional reports

### Current challenge in the management of preterm labor peculiar in Japan

In Japan, long-term tocolysis using ritodrine or/and MgSO_4_ until 34–36 weeks of gestation has often been performed for preterm labor. For example, among all patients with preterm labor, 28.7% have been reported to receive intravenous infusion of ritodrine for ≥ 28 days (38). The incidence of preterm birth in Japan has not been high (5–6%); however, recently there have been some observations concerning the adverse effects of long-term administration of tocolytic agents on mothers and children. According to a Japanese nationwide retrospective cohort study performed in 2014 (39), the occurrence of neonatal hyperkalemia was associated with concomitant usage of ritodrine and MgSO_4_ compared with no usage. In addition, the occurrence of neonatal hypoglycemia was associated with ritodrine of cessation directly before delivery. Another nationwide study indicated that a high proportion of maternal adverse effects such as lung edema, granulocytopenia, and rhabdomyolysis among patients treated with long-term usage of ritodrine (38). Therefore, in Japan, there are now in the process of changing from long-term tocolysis to short-term tocolysis as in Western countries.

### Preterm birth in Australia and New Zealand

In 2019, there were 303,054 babies born to 298,567 mothers in Australia, with an average maternal age of 30.8 years. 42.5% of women experienced spontaneous labor, 34.7% were induced and 22.5% had no labor. The main reasons for labor induction were diabetes, pre-labor rupture of membranes, and prolonged pregnancy. In 2019, the PTB rate was 8.6% representing 25,933 babies. The maternal mortality rate in 2019 was 6.4 deaths per 100,000 women giving birth (15, 16). There were 59,661 women giving birth in New Zealand during 2017 representing 60,026 live-born babies. 62.7% of women experienced spontaneous vaginal birth, 9.5% assisted birth, and 27.9% Cesarean section. In 2017, a total of 4,503 (7.5%) of babies were born preterm: 777 (1.3%) were born at under 32 weeks' gestation and 3,726 (6.2%) were born at 32–36 weeks' gestation. The most of live-born babies (91.5%) was within the normal weight range at birth (2.5–4.4 kg). A further 6.1% of babies were born with low birth weight (<2.5 kg) and 2.4% were born with a high birth weight (≥4.5 kg). In 2018, the fetal death rate was 6.8 per 1,000 total births and an infant death rate of 4.0 per 1,000 live births (15).

### Burden of preterm in India and discovery of molecular signatures for early detection

Group of Advanced Outcome and Research a DBT India initiative (Garbh-ini), an observational tertiary hospital-based pregnancy cohort in the North of India, funded by the Department of Biotechnology, Ministry of Science and Technology, was presented to discovery of molecular signatures of PTB using integrated multi-omic approaches. The cohort has reported proteomic changes in maternal saliva, and high vaginal fluid obtained serially along pregnancy, using the state-of-the-art label-free proteomics (SWATH-MS) approach. Detailed protein-protein interaction network analysis has revealed a list of proteins associated with maternal immune modulation, metabolism, host defense mechanism and tissue remodeling, organ development microbial defense in salivary and high vaginal fluid analysis respectively (40, 41). Toward mechanistic studies, placental derived exosomes isolated from maternal plasma sampled at three different trimesters and delivery were profiled across pregnancy for their protein cargo. Computational analysis of differentially expressed proteins revealed consistent upregulation of inflammatory pathways in term and preterm delivery mothers, upregulation of epithelial-mesenchymal transition pathways in term, and downregulation of coagulation/complement activation in PTB samples (42).

## Summary

In this report, we explored the current situation, research hotspots, and therapy strategies in China, Korea, Japan, Australia, and India. Despite the medical technology becoming more advanced in the last few decades, preterm birth rate had continued to increase from 9.8% in 2000 to 10.6% in 2014 globally, which equates to an estimated 14.84 million live preterm births in 2014. The authoritative statistics of “Top 10 countries for the number of preterm births in 2014” showed that four of the top 5 countries are from Asia and they accounted for 6.6 million (44.6%) of preterm births globally. This places a heavy psychological and financial burden on the families of preterm infants and associates with high socio-economic costs to the healthcare system. The goal for healthcare professionals and governments is to improve the above situation of preterm birth, reduce preterm birth rate and prevent potential consequences of preterm birth. However, high-level evidence-based clinical and basic researches to assist the community medical institutions and primary care givers to standardize diagnosis and treatment for preterm birth is inadequate, which may be the most needed scientific improvements in the Australasian context. The scarce high-quality preterm birth research is partly attributable to varieties of challenges related to study training, support, funding, ethics committees, effective health management systems as well as local collaboration. Therefore, global collaborations and multihospital and community cohorts need to be established for validation of the molecular signatures that have to date been associated with PTB. A multidisciplinary global approach in search of molecular and or clinical markers is required. Development and validation of predictive tools for preterm birth that could be implemented in a clinical setting would be a step further in reducing the rate of PTB and its related complications post-birth both in mother and child.

## Author contributions

NZ had full access to all the manuscript and had final responsibility for the decision to submit for publication. All authors designed the manuscript, collected data, revised, and approved the report.

## Conflict of interest

The authors declare that the research was conducted in the absence of any commercial or financial relationships that could be construed as a potential conflict of interest.

## Publisher's note

All claims expressed in this article are solely those of the authors and do not necessarily represent those of their affiliated organizations, or those of the publisher, the editors and the reviewers. Any product that may be evaluated in this article, or claim that may be made by its manufacturer, is not guaranteed or endorsed by the publisher.

## Author disclaimer

The article represents the views of the authors only, and not the views of their institutions.
